# Vincristine, Irinotecan, and Temozolomide in Patients With Relapsed/Refractory Neuroblastoma

**DOI:** 10.3389/fonc.2022.804310

**Published:** 2022-03-09

**Authors:** Jia Zhu, Juan Wang, Feifei Sun, Zijun Zhen, Tingting Chen, Suying Lu, Junting Huang, Yizhuo Zhang, Xiaofei Sun

**Affiliations:** ^1^Collaborative Innovation Center for Cancer Medicine, State Key Laboratory of Oncology in South China, Guangzhou, China; ^2^Department of Pediatric Oncology, Sun Yat-sen University Cancer Center, Guangzhou, China

**Keywords:** neuroblastoma, relapse, refractory, vincristine, irinotecan, temozolomide, efficacy, toxicity

## Abstract

**Purpose:**

The combination of irinotecan, temozolomide and vincristine has been proposed as an effective salvage regimen for some pediatric malignancies. Thus, we sought to evaluate this combination for patients with relapsed and refractory neuroblastoma (NB).

**Patients and Methods:**

In this retrospective study, forty-six patients with relapsed or refractory NB were treated with the combination of vincristine (1.5 mg/m^2^ i.v. day 1), irinotecan (50 mg/m^2^/day i.v. days 1–5) and temozolomide (100 mg/m^2^/day p.o. days 1–5) (VIT) during the period 2011–2019. All toxicities were documented.

**Results:**

A total of 251 cycles (median 6 cycles/patient) were administered. A complete response (CR) was achieved in 5 patients, partial response (PR) in 27 patients, stable disease (SD) in 8 patients, and progression disease (PD) in 6 patients, with an overall objective response rate (CR+PR) of 69.6%. Eighteen patients developed diarrhea with Grade 3 or less. Grade 1-2 hematologic toxicity occurred in 10 patients. Grade 3-4 hematologic toxicity developed in 32 patients. VIT was an effective regimen for different metastatic sites. UGT1A*28 genotyping performed in 7 patients revealed wild type. Diarrhea occurred in 4 of them.

**Conclusion:**

The shorter, 5-day VIT regimen is an active and well-tolerated salvage regimen in relapse/refractory NB.

## Introduction

Neuroblastoma is the most common extracranial solid tumor in childhood. Although most of these patients were sensitive to chemotherapy, the long-term outcome of high-risk patients has remained dismal with 5-year overall survival (OS) rates less than 50%. Salvage therapy could prolong survival in multiply relapsed patients. Patients with relapsed or recurrent neuroblastoma at the Sun Yat-Sen University Cancer Center received chemotherapy comprising vincristine, irinotecan, and temozolomide (VIT therapy) from 2011 as salvage chemotherapy. In this study we retrospectively analyzed the therapeutic effects, and toxicity of VIT in patients with relapsed or refractory neuroblastoma.

## Materials and Methods

### Patients and Treatment

We retrospectively retrieved information from the medical charts of patients treated in Sun Yat-sen University Cancer Center between June 1, 2011 and March 31, 2019, who experienced relapse or progression after first-line therapy. All of them received a combination regimen of vincristine, irinotecan, and temozolomide (VIT) as the salvage therapy. VIT comprised irinotecan 50 mg/m^2^ intravenously (IV) on days 1–5 (250 mg/m^2^/course), temozolomide 100 mg/m^2^/dose orally on days 1–5 approximately 1 hour before irinotecan administration, and vincristine 0.05 mg/kg or 1.5 mg/m^2^ (maximum dose 2 mg) IV over one minute on day 1 for 21 days per cycle. For patients unable to swallow capsules, temozolomide was allowed to mix with apple sauce or juice in 100 ml. Every cycle was repeated at three-week interval. Patients received atropine 0.01mg/kg subcutaneously 30 minutes before irinotecan administration. Cefixime was administered for patients who experienced ≥grade 3 diarrhea before next chemotherapy cycle. Loperamide was started immediately after >grade 2 diarrhea occurred as follows: 2mg at initial dose followed by 1mg every 2 hours thereafter for patents younger than 6 years of age; 4mg at initial dose, followed by 2mg, every 2 hours thereafter for patents older than 6 years of age. Loperamide continued to be administered at 6 doses after diarrhea improved. Total courses didn’t exceed 48 hours. For each patient, we collated demographic, disease-related, treatment-related and outcome data, such as treatment course, acute and late toxicity, disease status, place of primary and metastatic tumor, regimen of chemotherapy, response, and clinical follow-up.

#### Assessment of Response

The response was evaluated using imaging technologies such as computed tomography or magnetic resonance imaging, bone marrow aspirates, serum neuron-specific enolase (NSE), the urine vanillic mandelic acid (VMA)-urine creatinine ratio and urinary homovanillic acid (HVA)- urine creatinine ratio every two courses and three weeks after completion of treatment. Recommendations of radiological assessment comprised computed tomography, magnetic resonance imaging, 18F-FDG PET/CT, 68Ga DOTATATE and SPECT. Bone marrow trephine was performed at initial diagnosis. If the examination was positive, we would perform bone marrow trephine after completion of VIT chemotherapy. 123I-MIBG scan was not available in our center during the period. Response Evaluation Criteria in Solid Tumors (RECIST) were used to evaluate tumor response at the end of cycles 2, 4, 6 and 8 according to World Health Organization (WHO) criteria. Response was defined as per COG criteria as follows: complete response (CR): the disappearance of all target lesions, partial response (PR): at least 50% decrease in all measurable lesions, progressive disease (PD): at least 25% increase in the size of any lesions or development of new lesions, stable disease (SD): 0 to <25% increase or decrease in lesions. Objective response rate (ORR) was defined as follows: number of (CR+PR)/total number of evaluable patients. Toxicity was graded according to NCI Common Terminology Criteria for Adverse Events (CTCAE), Version 4.0.

### Statistical Analysis

Overall survival after relapse/progression was defined from the date of the first cycle of VIT to death from any cause, or censored at the date of last follow-up for the patients who were alive. Progression-free survival (PFS) was defined as the interval between the beginning of VIT treatment until treatment failure, death, treatment discontinuation for any reason, or detection of second neoplasm. Relapse-free survival (RFS) was defined as the interval between the status of complete response achieved by VIT until treatment failure, death, treatment discontinuation for any reason, or detection of second neoplasm. The RFS and PFS were calculated with the Kaplan–Meier method by using SPSS Statistics, version 25.0 (IBM Corp., Armonk, NY, USA). All tests were two-tailed and P < 0.05 was considered statistically significant.

### Ethical Approval

The present study was approved by the Ethics Board of the Sun Yat-sen University Cancer Center and conducted in accordance with the Helsinki Declaration. All original data were deposited on http://www.researchdata.org.cn (RDD number RDDA2019001318).

## Results

### Patient Characteristics

At SYSUCC, 46 children received VIT as salvage chemotherapy in refractory/relapsed (PD/Re) setting. There were 29 boys (63.0%) and 17 girls (37.0%) with a median age of 47.6 (range, 16.0 – 145.0) months. The International Neuroblastoma Staging System stage distribution was as follows: stage II: 1(2.2%) patient, stage III: 8 (17.4%) patients and stage IV: 37 (80.4%) patients. Five patients were stratified into intermediate-risk group and 41 were categorized into high-risk group at initial diagnosis on the basis of the following factors: stage, age, International Neuroblastoma Pathologic Classification, amplification of the *MYCN* oncogene within tumor tissue.

Thirty-five patients underwent MYCN amplification test on tumor specimens. MYCN amplification occurred in 8 patients and 27 patients were in the absence of MYCN amplification. Forty-six patients received first line therapy of vincristine/doxorubicin/cyclophosphamide (CAV) and ifosfamide/etoposide/cisplatin (VIP) at initial diagnosis. The median interval from the beginning date of initial diagnosis to the date of first relapse/progression was 19.2 (range, 3.0 – 47.7) months. Relapses or progression in the primary tumor occurred in 3 patients. Sites of metastases at relapse/progression were as follows: bone marrow/bone, lymph nodes, brain, mediastinum and abdomen. Patient characteristics are shown in **Table 1**.

**Table 1:** Patient characteristics (n=46).

Category	No. of Patients
Total	46
Age at enrollment, m, median(range)	47.6 (16.0~145.0)
Time to first PD, m, median(range)	19.2 (3.0~47.7)
Sex	
Male	29
Female	17
Stage at initial diagnosis	
II	1
III	8
IV	37
Risk stratification	
Intermediate-risk	5
High-risk	41
*MYCN* gene amplification	
Yes	8
No	27
Unknown	11
Location of recurrence/PD	
Mediastinum	6
Abdomen	6
Brain	7
Bone marrow/bone	15
Lymph node	9
Multiple sites	3

### Response to VIT

A total of 251 cycles were administered to 46 eligible patients (median of 6 cycles, range 2-10). VIT was used as first-line salvage therapy in 6 patients, second-line in 33 patients, third-line in 6 patients and fourth-line in 1 patient after progression or recurrence (**Table 2**). Overall response to VIT was assessed at the end of chemotherapy. Among all patients, 5 had complete response, 27 had partial response, 8 had SD, and 6 had progressive disease (**Table 2**). The objective response was 69.5% (complete response + partial response). The best response to VIT was achieved at the end of 4 courses for the median number of responders. During the treatment, one patient continued to have response in his lesions after 9 cycles. Further, among patients with different site involved, response rates to VIT regimen were as follows: mediastinum 100.0%, bone marrow/bone 80.0%, lymph node 77.8%, abdomen 50.0% and brain 42.9%, respectively. Thirty-four of the 46 patients (73.9%) had best responses (**Table 3**). In the patients who received VIT as first line, second line, third line and forth line salvage chemotherapy, the best response rates were 4/6, 24/33, 5/6 and 1/1, respectively (**Table 4**).

**Table 2:** Response to VIT chemotherapy for patients with refractory/relapsed neuroblastoma.

Variables	Number
Total VIT courses	251
Median VIT course	6 (2-10)
Cycles of VIT at best response to VIT	
Median	4
VIT line for refractory/relapsed neuroblastoma	
First-line	6
Second-line	33
Third-line	6
Fourth-line	1
Overall responses after VIT therapy	
CR	5
PR	27
SD	8
PD	6

VIT, vincristine, irinotecan, and temozolomide; CR, complete response; PR, partial response; SD, stable disease; PD, progression disease.

**Table 3:** Response to VIT chemotherapy based on sites at relapse/progression.

Sites	No. of patients	Best response				ORR
		CR	PR	SD	PD	(%)
Mediastinum	6	1	5	0	0	100
Abdomen	6	2	1	3	0	50.0
Brain	7	0	3	1	3	42.9
Bone marrow	15	4	8	3	0	80.0
Lymph nodes	9	0	7	1	1	77.8
Multiple sites (≥3 sites)	3	1	2	0	0	100
**Total**	**46**	**8**	**26**	**8**	**4**	**73.9**

VIT, vincristine, irinotecan and temozolomide; CR, complete response; PR, partial response; PD, progressive disease; SD, stable disease; ORR, objective response rate.

Regarding clinical benefit, VIT has shown the best response rate (illustrated in the bold values) in the whole cohort regardless of involved sites.

**Table 4:** Response to VIT chemotherapy as salvage regimen after relapse or progression.

VIT line	No. of patients	Best response				ORR
		CR	PR	SD	PD	(%)
First	6	0	4	0	2	66.7
Second	33	6	18	8	1	72.7
Third	6	2	3	0	1	83.3
Fourth	1	0	1	0	0	–

VIT, vincristine, irinotecan and temozolomide; CR, complete response; PR, partial response; PD, progressive disease; SD, stable disease; ORR, objective response rate.

### Toxicity

Toxicity was evaluated for the whole cohort. All toxicity results related to VIT chemotherapy were graded using the National Cancer Institute Common Terminology Criteria for Adverse Events version. Regarding neutropenia, toxicities included three patients with grade 1, seven with grade 2, eighteen with grade 3 and fourteen with grade 4, respectively. Grade 3–4 thrombocytopenia occurred in six patients (**Table 5**). Otherwise, non-haematologic toxicity observed included abdominal pain, diarrhea, nausea, vomiting, constipation and oral mucositis. Abdominal pain and diarrhea were the most common non-hematological adverse events. Toxicities are detailed in **Table 5**.

**Table 5:** Hematological and non-hematological toxicities.

Toxicity	Number
Neutropenia	
0	4
1	3
2	7
3	18
4	14
Thrombocytopenia	
0	25
1	12
2	3
3	3
4	3
Abdominal pain/diarrhea	
0	28
1	10
2	5
3	3
Nausea	
0	28
1	15
2	3
Constipation	
0	39
1	5
2	2
Vomiting	
0	33
1	8
2	3
3	2
Oral mucositis	
0	44
1	1
2	1

### Association of UGT1A1(UDP-Glycosyltransferase 1 Polypeptide A1) Genotyping and Diarrhea

The UGT1A1*28 promoter polymorphism was genotyped by PCR amplification in seven patients. Analysis of the UGT1A1 gene revealed that the seven patients possessed a wild-type. Four patients experienced diarrheas. Two patients carried UGT1A1*6 heterozygous genotypes. One patient developed diarrhea.

### Progression Free Survival After First VIT Therapy

**Figure 1:**
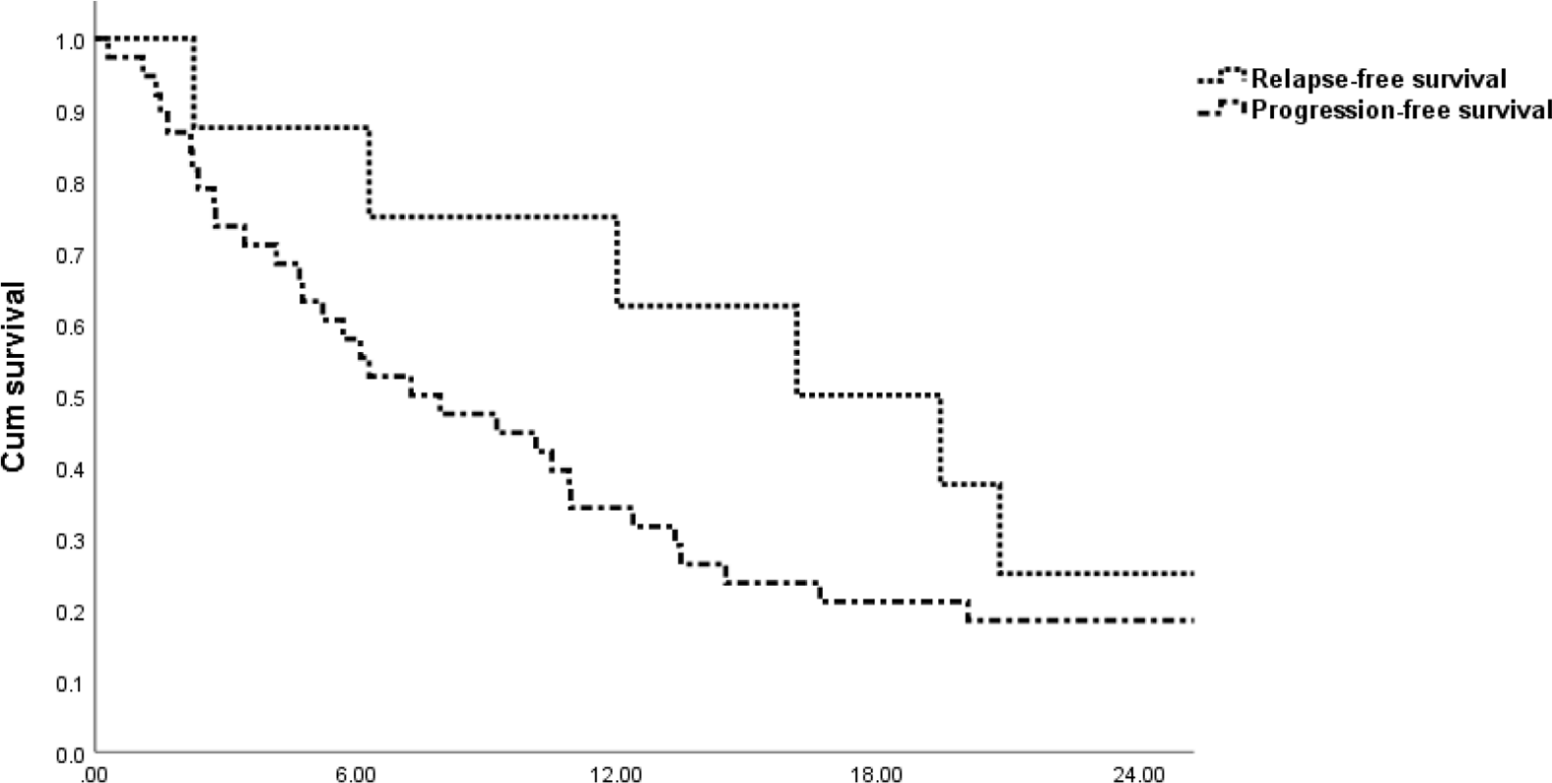
Survival rates from the initiation of VIT to progression or relapse. (months).

The median follow-up time was 43.9 months (range: 17.8–133.6 months) for all patients. The median RFS time and PFS time from the beginning of VIT treatment for the whole cohort were 16.1 (range, 2.3–32.0) months and 7.3 (range, 0.3-50.7) months, respectively (**Figure 1**).

## Discussion

In our center, treatment for our patients with neuroblastoma is based on the Children’s Oncology Group protocol. Such patients received risk-stratified therapy. First-line adjuvant chemotherapy with cycles of vincristine, doxorubicin, cyclophosphamide alternating with cycles of ifosfamide, cisplatin and etoposide was administered to high-risk patients. First-line treatment included only standard dose chemotherapy courses. Each cycle was detailed as follows: vincristine 1.5 mg/m^2^ (2 mg maximum total dose) intravenously on days 1, doxorubicin 50 mg/m^2^ intravenously on day 1, cyclophosphamide 1 g/m^2^ intravenously on days 1-2; ifosfamide 1.5 g/m^2^ intravenously on days 1-4, cisplatin 25 mg/m^2^ intravenously on days 1-4, etoposide 100 mg/m^2^ intravenously on days 1-4. Every cycle was repeated at the 3-week interval. Total cycles of chemotherapy were 8 courses. Although the event-free survival of high-risk patients is still dismal, they could still achieve CR after salvage therapy. In the absence of anti-disialoganglioside immunotherapy, overall survival rates have improved owing to progress in multimodality treatment. The combination of irinotecan, and temozolomide has shown activity against relapsed or refractory neuroblastoma (1).In 19 refractory NB patients treated with VIT, 2 had CR and 7 had object responses with an objective response (CR+PR) of 47.4%. In 17 patients with progressive disease, one had PR and two had objective responses.

As a second generation alkylating agent, temozolomide is prodrug of MTIC (3-methyl-(triazen-1-yl)imidazole-4-carboxamide). Oral temozolomide has demonstrated activity for pediatric rhabdomyosarcoma (2–4). Irinotecan is a camptothecin prodrug which is converted by endogenous carboxylesterases to SN-38, a topoisomerase-I inhibitor causing inhibition of both DNA replication and transcription. The combination of temozolomide and irinotecan has shown synergistic activity against PD/Re pediatric solid tumors (5). The synergy can be explained due to DNA methylation by temozolomide and recruitment of topoisomerase I cleavage complexes, potentiating irinotecan to stabilize the DNA–enzyme complex that caused cytotoxicity of the tumor cells (6). Irinotecan is administered intravenously on days 1-5 approximately one hour after temozolomide intake in order to maximize the synergistic effect (7). Temozolomide was obtained commercially in the form of capsules. Patients were allowed to open them and mix with apple sauce or juice because of steady state in acidic environment and oral mucositis. Irinotecan was administered intravenously over 90 minutes at the dose of 50mg/m^2^.

The combination of irinotecan and temozolomide(IT) has demonstrated activity in various types of PD/Re solid tumors. Based on preclinical data, irinotecan was administered as protracted regimen as follows: 20mg/m^2^, 5 days per week for 2 consecutive weeks (daily×5×2). VIT was administered in shortened regimen every 21 days as follows: vincristine, 1.5 mg/m^2^ intravenously (IV) on day 1; irinotecan, 50mg/m^2^ IV, days 1–5; temozolomide 100mg/m^2^ orally, days1–5. Vincristine (1.5mg/m^2^, max 2mg). Study exploring irinotecan administration have shown that shorter course of 50mg/m^2^/day×5 days on a 3-week schedule is equivalent to the protracted schedule (6). Although several studies have shown that there was difference in efficacy between protracted regimen and shortened regimen, more data evaluating the efficacy and safety of the two regimens shows no significant difference in response rates or toxicity (7). We utilized shortened regimen on 3-week interval for patient convenience. All patients were heavily pretreated with multiagent regimen that includes doxorubicin. Irinotecan was less influenced by P-glycoprotein multi-drug resistance compared with topoisomerase II inhibitors such as doxorubicin and etoposide (8). Irinotecan has shown antitumour activity in preclinical models of topotecan-resistant xenografts (8). Previous study with small sample size demonstrated that the number of previous treatment regimens received did not affect the outcome of those who received VIT as salvage therapy (6). An optimal responses were seen in patients who used VIT as first, second or third line of therapy after relapse or progression (**Table 4**). VIT did not show significant difference in response rates among different metastatic sites (**Table 5**). Treatment directed against pediatric medulloblastoma has been shown activity in preclinical mouse models because of its blood brain barrier penetration. One patient with brain metastasis localized to the parenchyma in our cohort responded well to chemotherapy with VIT, resulting in CR in the bone marrow and disease-free survival of 8 years after multidisciplinary treatment. There is synergistic activity, efficient penetration through the blood–brain barrier as well as no cross-resistance, and hence therapy of VIT could benefit PD/Re patients with neuroblastoma.

Both preclinical and clinical trials showed activity of VIT in a variety of pediatric cancers. VIT regimen was used as third-line chemotherapy in 34 patients with PD/Re solid tumors including 8 patients with neuroblastoma (9). Salvage chemotherapy prior to VIT comprised ifosfamide/etoposide and ifosfamide/carboplatin/etoposide. Among the 8 patients, 1 had complete response, 1 had partial response, 3 had stable disease, and 3 had progressive disease. Median progression free time was three months in all NB patients (9). The overall objective response (complete response + partial response) in our cohort was 69.6% (34/46) with 7.3 months median progression free time which may have contributed to raise the opportunity for local control. The combination of irinotecan and temozolomide (IT) have been used for relapsed/refractory neuroblastoma in previous study. The combination of IT produced objective responses of 33% and 15%, respectively (1, 10). Vincristine in combination with IT have shown synergistic activity in patients with rhabdomyosarcoma (11). Our study has demonstrated better ORR than results in previous study likely due to the addition of vincristine to the IT backbone.

The shortened schedule of irinotecan resulted in less grade 3–4 toxicity. A trial comparing protracted versus short schedule of irinotecan combined with vincristine and temozolomide showed higher frequency of dose limiting toxicity in the protracted regimen (12). Temozolomide has relatively minor myelosuppressive side effect and its dose limiting toxicity is mainly neutropenia and thrombocytopenia. Dose-limiting toxicities of irinotecan are primarily diarrhea and abdominal pain, accounting for 20% of patients (9). Overall the toxicities observed in this study were diarrhea and abdominal pain, accounting for 39.1%. Irinotecan could induce cholinergic syndrome, characterized by diarrhea, vomiting and abdominal pain. Irinotecan-related cholinergic syndrome is managed with atropine in prophylaxis. Irinotecan is metabolized by a specific route into SN38. Enteric bacteria cleave the drug-glucuronide bond in SN38. The resultant free SN38 metabolite causes diarrhea through its direct effect on the intestinal mucosa. Cefixime was used to eliminate intestinal bacteria and reduce irinotecan associated diarrhea. Cefixime 8mg/kg daily (maximum 400mg) was administered starting 2 days before each cycle for patients who experienced >grade 2 diarrhea in last cycles (13). Loperamide and hydration support were administered to patients with diarrhea. Eighteen patients developed diarrhea among 46 patients. No >grade 3 diarrhea occurred in this cohort. Patients treated on our study did not experience severe intestinal obstruction and neuropathy. Vincristine was omitted for patients with >grade 3 neuropathy (14). Myelosuppression associated with VIT regimen was mild. Myelosuppression was manageable, with eighteen patients experiencing grade 3 and fourteen developing grade 4 myelosuppression. These heavily pretreated patients tolerated VIT regimen after first line of intense therapy. VIT regimens were readily administered in the outpatient setting and hence had the benefits of shorter duration of total hospital days. A few clinical trials have explored IT regimen in combination with other cytotoxic therapies to improve outcomes. For example, the addition of vincristine or bevacizumab to IT exhibited better anti-tumor activity and associated with more durable response than irinotecan alone (15). In addition, activity is further improved when synergistic combinations of IT regimen and radiotherapy were used.

Irinotecan is converted by endogenous carboxylesterases to metabolite SN-38. UGT1A1 is responsible for detoxification of SN-38 through the process of glucuronidation. Expression of UGT1A1 is, in part, under the control of a polymorphic dinucleotide repeat sequence within the UGT1A1 promoter TATA box, varying from five to eight copies of a TA repeat([TA]nTAA) (16). The (TA) 6 TAA allele is considered wild-type. The UGT1A1∗ 28 allele consisting of 7 TA repeats is the most frequently variant type. Homozygous UGT1A1 ∗ 28 polymorphism is associated with a reduction of about 80% of UGT1A1 transcription. Reduced UGT1A1 gene expression leads to a decrease of inactive SN-38G and hence increases irinotecan-related toxicity. There was no clear relationship observed between UGT1A1 genotype and irinotecan-induced toxicity in adult. Blood samples for UGT1A1*28 genotyping were collected from 7 children in our study. All patients carried the UGT1A1*28 wild (6/6) genotype. We are unable to draw a definite conclusion of association between UGT1A1*28 genotype and irinotecan-related diarrhea in the limited number of patients. Factors other than UGT1A1*28 genotype may be responsible for irinotecan-induced diarrhea (13).

In conclusion, the VIT regimen had an objective response of 69.6% in heavily pretreated refractory/relapsed neuroblastoma. Among the 46 evaluable patients, the best response to VIT was achieved at the median of 4 cycles. The 5-day VIT regimen is an active and well-tolerated salvage regimen in relapse/refractory NB with manageable toxicities. Vomiting induced by oral temozolomide is another concern. The study can also serve as a template for intravenous temozolomide to the vincristine, irinotecan chemotherapy backbone in the future.

## Data Availability Statement

The raw data supporting the conclusions of this article will be made available by the authors, without undue reservation.

## Ethics Statement

The studies involving human participants were reviewed and approved by the Ethics Board of the Sun Yat-sen University Cancer Center. Written informed consent to participate in this study was provided by the participants’ legal guardian/next of kin.

## Author Contributions

JZ, XS, and YZ conceived and designed the analysis. TC and JW collected the data. JH, SL, and FS contributed data and analysis tools. ZZ performed the analysis. JZ, TC, and ZZ wrote the paper. All authors contributed to the article and approved the submitted version.

## Conflict of Interest

The authors declare that the research was conducted in the absence of any commercial or financial relationships that could be construed as a potential conflict of interest.

## Publisher’s Note

All claims expressed in this article are solely those of the authors and do not necessarily represent those of their affiliated organizations, or those of the publisher, the editors and the reviewers. Any product that may be evaluated in this article, or claim that may be made by its manufacturer, is not guaranteed or endorsed by the publisher.
